# Kurative Strahlentherapie von Oligometastasen: Langzeitergebnisse der SABR-COMET-Studie

**DOI:** 10.1007/s00066-021-01745-w

**Published:** 2021-02-02

**Authors:** Jürgen Dunst

**Affiliations:** Klinik für Strahlentherapie, Campus Kiel, Universitätsklinikum Schleswig-Holstein, Feldstr. 21, 24105 Kiel, Deutschland

## Hintergrund

Vor etwa 25 Jahren wurde erstmals durch die Radioonkologen Hellman und Weichselbaum die Hypothese der Oligometastasierung formuliert [2]. Darin wird postuliert, dass Patienten mit wenigen Metastasen (also einem frühen Stadium IV einer Erkrankung) nicht nur eine makroskopische, sondern vermutlich auch eine mikroskopische Tumorlast haben, die etwa einem Stadium III oder sogar II entspricht. Die Idee war, diese Patienten dann auch wie ein Stadium III zu behandeln, also mit einer „kurativen“ lokalen Therapie und „adjuvanten“ medikamentösen Therapie.

So einfach diese Theorie auch erscheinen mag, die Umsetzung in der Klinik und die Durchführung von Studien waren schwierig. Dazu braucht man nämlich neben einer suffizienten und schnellen Bildgebung vor allem eine lokale Therapie, die effektiv, gut verträglich und problemlos mit der (leitliniengerechten) medikamentösen Therapie kombinierbar ist. Mit den modernen hochpräzisen Bestrahlungsmethoden steht eine solche Technik seit einigen Jahren zur Verfügung. Komplizierend kam und kommt aber hinzu, dass die rasante Entwicklung neuer medikamentöser Therapieformen, z. B. zielgerichteter Therapien oder Immuntherapien, das interdisziplinäre Interesse an Studien mit lokalen Therapieformen eher zurückgedrängt hat. Bisher gibt es aber immerhin schon mehrere randomisierte Studien, die alle einheitlich eine teilweise eindrucksvolle Überlebensverbesserung durch die additive lokale Therapie zeigen und damit die Oligometastasenhypothese belegen [1, 3, 5, 8]. Die erste publizierte Studie war die SABR-COMET-Studie [6], deren Langzeitdaten jetzt veröffentlicht wurden [7].

## Patientenkollektiv und Methodik

In die randomisierte Phase-II-Studie konnten Patienten eingeschlossen werden, die 1–5 Metastasen bei kontrolliertem Primärtumor aufwiesen. Alle Metastasen mussten für eine hoch dosierte stereotaktische Strahlentherapie („stereotactic ablative body radiotherapy“ [SABR]) geeignet sein. Nach 1:2-Randomisation erhielten Patienten im Kontrollarm eine leitliniengerechte Systemtherapie; eine palliative Strahlentherapie symptomatischer Metastasen war erlaubt. Im experimentellen Arm wurden zusätzlich zur Systemtherapie alle Metastasen hoch dosiert bestrahlt („comprehensive treatment of oligometastases“ [COMET]); typische Regime waren Einzeitbestrahlungen mit 16–24 Gy oder 3–8 Fraktionen mit 30–60 Gy Gesamtdosis. Primärer Endpunkt war das Gesamtüberleben.

## Ergebnisse

Von 2012 bis 2016 wurden 99 Patienten an 10 Prüfzentren randomisiert. Die Primärtumoren waren Mammakarzinome, Bronchialkarzinome und kolorektale Karzinome (*N* jeweils 18), Prostatakarzinome (*N* = 16) und andere Entitäten (*N* = 29). Das mediane Follow-up lag bei 51 Monaten. Es gab einen signifikanten Vorteil im Gesamtüberleben (OS) im experimentellen Arm gegenüber der Kontrolle, und zwar sowohl im 5‑J-OS mit 42,3 vs. 17,7 % und im medianen OS mit 50 vs. 28 Monate (HR = 0,47, *p* = 0,006) als auch im progressionsfreien Überleben (5-J-PFS 17,3 % vs. 0 %, medianes PFS 11,6 vs. 5,4 Monate, *p* = 0,001). Der Überlebensvorteil blieb in einer Post-hoc-Analyse, in der Patienten mit Prostatakarzinom ausgeschlossen wurden, gleich groß (5-J-OS 33,1 % vs. 16,2 %). Unerwünschte Ereignisse ≥ Grad 2 waren in der ersten Publikation im Jahr 2019 bei 29 % der Patienten im experimentellen Arm vs. 9 % im Kontrollarm beobachtet worden; neue AE waren seitdem nicht aufgetreten. In der Lebensqualität gab es zwischen den beiden Armen keine Unterschiede.

## Schlussfolgerungen der Autoren

Der bereits in der ersten Publikation der Studie nachgewiesene Überlebensvorteil im experimentellen Therapiearm hat sich mit längerer Nachbeobachtungszeit stabilisiert und vergrößert. Die additive hoch dosierte Strahlentherapie der Oligometastasen war gut verträglich und hatte keinen negativen Effekt auf die Lebensqualität.

## Kommentar

Der SABR-COMET-Trial kann nach meiner Einschätzung trotz des Designs (Phase II analog AMG) und der geringen Patientenzahl durchaus als „milestone study“ angesehen werden. Die absolute Differenz im Gesamtüberleben beträgt 25 %-Punkte; das ist im Stadium IV beachtlich und kann sich bei diesem Kollektiv durchaus messen mit den weltweit als Durchbruch gefeierten Erfolgen der Immuntherapie. Die additive Strahlentherapie war gut verträglich. Und andere Studien bestätigen die Daten. Es wird sicher noch etwas dauern, bis diese zielgerichtete Strahlentherapie von Oligometastasen in Leitlinien verankert wird. Bisher haben deshalb die Radioonkologen international diese Therapie zwar zunehmend eingesetzt, aber doch mit einer gewissen Zurückhaltung [4]. Zunehmend muss man sich als behandelnder Arzt aber fragen, inwieweit es ethisch vertretbar ist, eine solche Therapie geeigneten Patienten vorzuenthalten.

Natürlich kann man Kritik üben, und einzelne Aspekte der Studie und der Ergebnisse sind in der Tat diskussionsbedürftig:

1. Der randomisierte SABR-COMET-Trial war als „proof of principle“ konzipiert, und die Zahl von 99 randomisierten Patienten reichte für ein signifikantes Ergebnis aus. Dennoch ist die Studie zu klein für ein „practice-changing“. Weitere größere Studien sind wünschenswert, auch wenn alle bisher verfügbaren Daten das Konzept der additiven Lokaltherapie bestätigen.
2. Das Patientenkollektiv war, wie bei einer Proof-of-principle-Studie üblich, recht heterogen. Es muss also genauer geklärt werden, ob es spezielle Tumorentitäten gibt oder Subgruppen, die unterschiedliche Effekte zeigen.
3. Die lokale Kontrolle der initialen Metastasen wurde durch die Strahlentherapie nur geringfügig verbessert. Die absolute Differenz bei der lokalen Kontrolle war nämlich zwischen beiden Therapiearmen geringer als die absolute Differenz im Gesamtüberleben, und sie war im experimentellen Arm mit 63 % verhältnismäßig schlecht. Für diesen Widerspruch könnten aus unserer Sicht zwei Erklärungen infrage kommen, nämlich erstens „Pseudoprogressionen“ nach Strahlentherapie oder zweitens abskopale Effekte. Nach hoch dosierter Strahlentherapie werden erfahrungsgemäß oft Pseudoprogressionen beobachtet, also Vergrößerungen von Läsionen in der Bildgebung, erhöhte KM-Aufnahmen oder Signalabnormalitäten im MRT. Gut bekannt ist dies bei der Radiochirurgie von Hirnmetastasen, aber auch bei der Behandlung von Lungen- und Lebermetastasen. Daher sind weitere Erfahrungen bei der Interpretation von Phänomenen der Bildgebung erforderlich. Außerdem sollte der Befunder die Bestrahlungsparameter kennen, insbesondere die Dosisverteilung, und wir als Radioonkologen sollten auf diese Phänomene in der Bildgebung hinweisen. Alternativ könnte man auch an abskopale Effekte denken, also eine Remissionsinduktion nicht bestrahlter Läsionen durch strahleninduzierte immunologische Effekte. Diese sind aus meiner Sicht allerdings weniger wahrscheinlich.
4. Während und nach der SABR-COMET-Studie sind zahlreiche neue Medikamente, insbesondere die Therapie mit PD-L1-Inhibitoren, in die Klinik eingeführt worden. Wie groß der Effekt der Strahlentherapie von Metastasen heute im Zeitalter der Immuntherapie wäre, bleibt vorerst noch unklar. Allerdings zeigen die Daten beispielsweise der randomisierten Pembro-RT-Phase-II-Studie ebenfalls eine Verbesserung des OS bei Strahlentherapie plus Pembrolizumab versus Pembrolizumab allein. Das mediane Überleben verhielt sich 15,9 vs. 7,6 Monate mit einer ähnlichen Hazard Ratio [9]. Es ist nach den aktuellen Daten also durchaus wahrscheinlich, dass der Effekt der Strahlentherapie auch bei aktuellen medikamentösen Therapieregimen annähernd gleich ist.

## Fazit

Die hoch dosierte kurativ intendierte Strahlentherapie von Oligometastasen ist für geeignete Patienten durchaus eine interessante Option.

*Jürgen Dunst, Kiel*
